# A Review of Steel Slag Carbonation: Mechanisms, Applications, and Sustainability Assessment

**DOI:** 10.3390/ma19020286

**Published:** 2026-01-09

**Authors:** Xinyue Liu, Xianbin Ai, Zhigang Que, Xiaoming Liu, Zengqi Zhang

**Affiliations:** 1 Institute of Energy Research, Jiangxi Academy of Sciences, Nanchang 330096, China; quezhigang@126.com; 2 School of Metallurgical and Ecological Engineering, University of Science and Technology Beijing, Beijing 100083, China; liuxy@ustb.edu.cn (X.L.); liuxm@ustb.edu.cn (X.L.); zhangzq@ustb.edu.cn (Z.Z.)

**Keywords:** steel slag, carbon capture and utilization, influencing factors, industrial application, environmental impact, economic cost

## Abstract

Steel slag (SS), as a major solid waste of the steel industry, has CO_2_ sequestration potential due to its rich calcium and magnesium alkaline components. SS carbonation is a promising strategy gaining industrial traction to simultaneously treat industrial solid waste and greenhouse gases. This article firstly describes the properties of SS and summarizes the research progress of SS carbonation. The classification of mineral carbonation technology is introduced, and the advantages and disadvantages are analyzed. The key factors affecting the SS carbonation are discussed. Then, the current industrial application status and life cycle assessment results are summarized. Finally, the conclusions are summarized, and the future research direction is proposed. Carbonation of SS can effectively fix CO_2_ and produce high-value-added products, realizing a win–win situation of environmental and economic benefits, which is of great significance to the green transformation of the steel industry and the realization of the “double carbon” goal.

## 1. Introduction

As a pillar industry of the national economy and an energy-intensive industry, the steel industry occupies a central position in the global industrial system. According to the World Steel Association, the global crude steel production was 1.885 billion tonnes in 2024, of which China’s crude steel production reached 1.005 billion tonnes [1], accounting for more than half of the global total. However, steel production comes with significant resource and environmental costs. Each ton of crude steel produced emits approximately 1.92 tonnes of CO_2_ [1]. The global steel industry’s carbon emissions exceed 3.6 billion tonnes annually, accounting for approximately 10% of total global carbon emissions [2]. China’s steel industry emits more than 1.9 billion tonnes of carbon dioxide per year, accounting for approximately 15% of the country’s total carbon emissions [3]. China has set the strategic goals of “carbon peaking” and “carbon neutrality” to cope with the continuous rise in CO_2_ concentration in the atmosphere, aiming to reach peak carbon emissions by 2030 and achieve carbon neutrality by 2060 [4].

The steel industry is not only one of the largest sources of CO_2_ emissions but also one of the largest sources of industrial solid waste. Steel slag (SS) is an alkaline industrial solid waste generated during steelmaking, with approximately 0.1–0.2 tonnes of slag emitted for every ton of crude steel produced [5,6,7]. Annual SS emissions in China exceed 160 million tonnes, with cumulative stockpiles reaching about 1.8 billion tonnes. The comprehensive utilization rate of SS remains below 30%, far lower than that of developed countries [8]. A small portion of SS is utilized in the production of cementitious materials and road base materials [9,10,11,12]. However, the majority of SS is simply stockpiled or directly landfilled. This not only consumes land resources but also potentially leads to the migration of heavy metals from the slag into soil, groundwater, and air over time due to weathering and erosion [13]. As the world’s largest steel producer, China not only faces the daunting task of reducing CO_2_ emissions but also urgently needs to develop resource utilization pathways for SS.

SS carbonation technology utilizes the abundant calcium or magnesium silicate minerals in SS to react with CO_2_ through carbonation, forming stable carbonate products [14,15,16]. This process not only achieves permanent CO_2_ sequestration, contributing to carbon neutrality goals, but also reduces the free calcium oxide (f-CaO) and free magnesium oxide (f-MgO) content in SS, which improves the volume stability of SS, enhancing its applicability in construction materials [17,18]. This offers a promising approach for the steel industry to reduce carbon emissions and recycle solid waste resources [19].

The efficient and economically viable SS carbonation technology is important for the sustainable development of the steel industry. This technology is not only a way to achieve carbon neutrality but also promotes the use of resources and waste recycling. Despite the remarkable research achievements in the past two decades, several obstacles hinder its widespread industrial application, including low carbonation efficiency under ambient conditions, high energy consumption for grinding and pressurization, challenges in product separation and purity control, and the lack of standardized protocols for carbonated product certification.

### Research Significance

Since 2020, several review papers on SS carbonation technology have been published, and each has a distinct focus. For instance, Baras et al. [20] reviewed key factors influencing SS carbonation, including particle size, temperature, time, CO_2_ pressure, and CO_2_ concentration and so on. Wang et al. [21] analyzed the effects of curing temperature, CO_2_ concentration, pressure, SS particle size, and leaching reagents on the actual CO_2_ uptake of SS and focused on the mechanism of accelerated carbonation on the mechanical properties and stability of SS-based building materials. Zhang et al. [22] comprehensively reviewed carbonation methods of SS and evaluation methods of carbonation sequestration, elucidated the effects of temperature, particle size, liquid-to-solid ratio, and CO_2_ pressure on SS carbonation. DiGiovanni et al. [23] reviewed the mechanisms of carbonation reactions and key parameters controlling the reaction process, along with applications of carbonation SS in binders or aggregates in the construction industry. Wang et al. [24] reviewed the carbonation mechanism, factors affecting the carbonation process, and the application of carbonated SS.

These reviews focus on exploring technical pathways and individual influencing factors of SS carbonation. There is a lack of systematic reviews that summarize its influencing factors, industrial demonstration cases and environmental and economic evaluations. Furthermore, microbial carbonation has not received sufficient attention in existing reviews. This paper clarifies the reaction mechanisms and advantages/disadvantages of direct dry carbonation, direct wet carbonation, indirect carbonation and microbial carbonation. It explores the complex relationships among key parameters, reviews industrial demonstration cases worldwide, and provides environmental and economic evaluations.

This review provides a comprehensive and critical analysis of the current status of SS carbonation technology. The characteristics of SS are first introduced. Then, the reaction mechanisms, advantages and disadvantages of the direct dry, direct wet, indirect and microbial carbonation are analyzed in depth. On this basis, the key factors affecting the carbonation conversion are systematically discussed. Next, industrial application studies of SS carbonation are reported to verify the feasibility of recovering high-value-added products. Then, the environmental impact and economic feasibility of the carbonation process are analyzed. Finally, the current research findings are summarized, and future research directions are proposed to accelerate the conversion of this technology from laboratory scale to industrial application.

## 2. The Composition of SS

### 2.1. Chemical Composition

**Figure 1:**
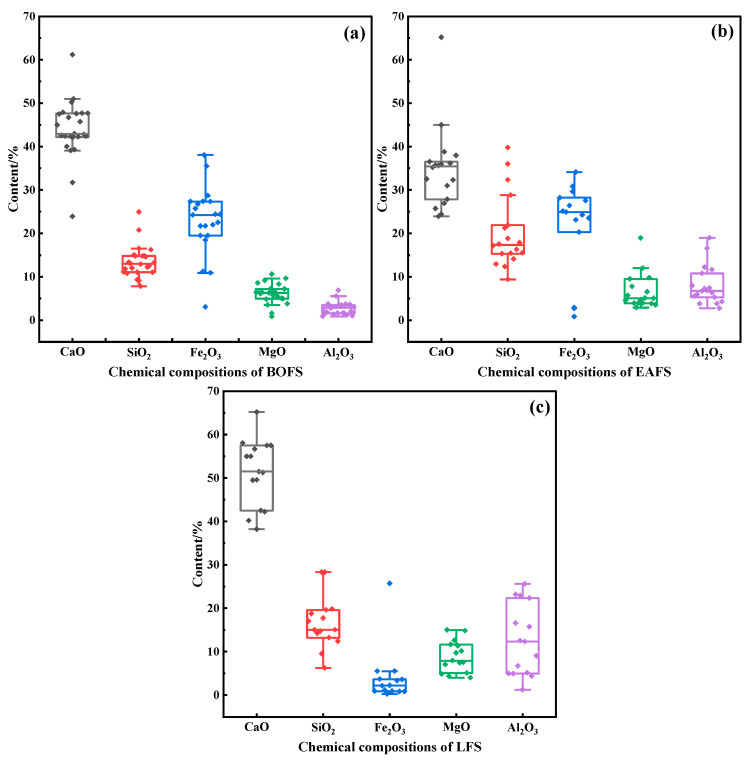
Main chemical compositions of SS (wt%): (**a**) BOFS; (**b**) EAFS; (**c**) LFS. Data sources are from literature [2,13,26,27,28,29,30,31,32,33,34,35,36,37,38,39,40,41,42].

Due to the different production conditions and raw material inputs in the process of steel production, SS is primarily categorized into basic oxygen furnace slag (BOFS), electric arc furnace slag (EAFS), and ladle furnace slag (LFS) [25]. The main chemical compositions are CaO, SiO_2_, Fe_2_O_3_, MgO and Al_2_O_3_ [2,6,26,27], and their contents are shown in Figure 1. It can be seen that LFS has the highest CaO content (median 51.5 wt%), followed by BOFS (median 42.9 wt%), and EAFS is the lowest (median 35.4 wt%). The SiO_2_ content is highest in EAFS, followed by LFS, and lowest in BOFS, though the median differences are less than 5 wt%. BOFS and EAFS exhibit high Fe_2_O_3_ content of around 24 wt% due to the intense oxidation of iron during primary smelting processes. In contrast, LFS has a very low Fe_2_O_3_ content, usually less than 5 wt%. The differences in the MgO content among the three slags are relatively small. LFS typically exhibits higher Al_2_O_3_ content, which may be attributed to the addition of aluminum-based deoxidizers during the production of certain specialty steels.

From the perspective of carbonation potential, LFS theoretically has the greatest carbon sequestration potential due to its highest CaO content and lowest FeO content. Although BOFS has a relatively high Fe_2_O_3_ content, its high alkalinity (CaO/SiO_2_ ratio) makes it a good raw material for the carbonation reaction. Whereas EAFS is low in CaO content and high in SiO_2_ and Fe_2_O_3_ content, and its carbonation conversion may be limited.

### 2.2. Mineral Composition

The mineral composition of SS has been studied extensively by researchers. Table 1 shows the minerals potentially present in SS. Calcium, as the primary element in SS, exhibits the most diverse forms, with main mineral phases being dicalcium silicate (Ca_2_SiO_4_, C_2_S in three main forms, α-C_2_S, β-C_2_S and γ-C_2_S) and tricalcium silicate (Ca_3_SiO_5_, C_3_S) [43]. Additionally, calcium may exist as free calcium oxide (f-CaO), portlandite (Ca(OH)_2_), calcite (CaCO_3_) and mayenite (12CaO·7Al_2_O_3_, C_12_A_7_). In addition to combining with calcium to form calcium silicate, silicon may also exist in quartz (SiO_2_) or form complex aluminosilicates with calcium, aluminum, and other elements, such as CaAl_2_SiO_6_, Ca_2_Al_2_Si_2_O_7_, and Ca_3_Al_2_(SiO_4_)_1.5_(OH)_0.5_. The mineral phases of iron mainly include FeO, Fe_2_O_3_, srebrodolskite (Ca_2_Fe_2_O_5_), tetracalcium ferroaluminate (4CaO·Al_2_O_3_·Fe_2_O_3_, C_4_AF), and the RO phase. Magnesium may be present in the RO phase and periclase (f-MgO), and also forms various silicate minerals, such as magnesium olivine (Mg_2_SiO_4_), merwinite (Ca_3_Mg(SiO_4_)_2_), monticellite (CaMgSiO_4_). Aluminum rarely forms distinct minerals in SS, primarily occurring as minor constituents in C_12_A_7_, Ca_2_(Al, Fe)_2_O_5_, or complex calcium aluminosilicate minerals.

## 3. Mechanisms of Steel Slag Carbonation

The conversion of CO_2_ into stable carbonates using SS can be achieved through various pathways, including direct dry carbonation, direct wet carbonation, indirect carbonation and microbial carbonation [48,49,50,51]. Each pathway has distinct reaction mechanisms, advantages and disadvantages, as summarized in Table 2. The selection of a pathway depends on multiple factors such as the available CO_2_ source, energy consumption, and desired characteristics of the final product. Regardless of the pathway chosen, the process is an exothermic reaction. Gaseous CO_2_, an acidic gas, reacts with the basic calcium and magnesium-bearing minerals in SS.

### 3.1. Direct Dry Carbonation

Direct dry carbonation is a process where SS particles react directly with CO_2_ gas to form stable carbonates, similar to natural weathering [52,53]. This is a gas–solid reaction. The chemical reactions of the SS mineral phases that may occur are shown in Equations (1)–(10) [21,54,55].

CaO (s) + CO_2_ (g) → CaCO_3_ (s) (1)

MgO (s) + CO_2_ (g) → MgCO_3_ (s) (2)

Ca(OH)_2_ (s) + CO_2_ (g) → CaCO_3_ (s) + H_2_O (l) (3)

Mg(OH)_2_ (s) + CO_2_ (g) → MgCO_3_ (s) + H_2_O (l) (4)

Ca_2_SiO_4_ (s) + 2CO_2_ (g) → 2CaCO_3_ (s) + SiO_2_ (s) (5)

Ca_3_SiO_5_ (s) + 3CO_2_ (g) → 3CaCO_3_ (s) + SiO_2_ (s) (6)

CaSiO_3_ (s) + CO_2_ (g) → CaCO_3_ (s) + SiO_2_ (s) (7)

Ca_3_Mg(SiO_4_)_2_ (s) + 4CO_2_ (g) → 3CaCO_3_ (s) + MgCO_3_ (s) + 2SiO_2_ (s) (8)

Mg_2_SiO_4_ (s) + 2CO_2_ (g) → 2MgCO_3_ (s) + SiO_2_ (s) (9)

Mg_3_Si_2_O_5_(OH)_4_ (s) + 3CO_2_ (g) → 3MgCO_3_ (s) + 2SiO_2_ (s) + 2H_2_O (l) (10)

For a porous slag particle, direct dry carbonation reaction is typically described using the unreacted core shrinkage model [56]. This model posits that the chemical reaction occurs at an interface that moves from the outer surface of the particle toward its center as the reaction proceeds. Formed products gradually coat the particle surface, creating a product layer. Subsequently, CO_2_ diffuses through this layer until reaching the unreacted core surface, where carbonation reactions occur, causing the core to progressively shrink [57,58]. The carbonation reaction primarily involves three steps:

(1) External Diffusion: Diffusion of CO_2_ gas from the bulk gas phase through the gas film surrounding the particle.
(2) Internal Diffusion: Diffusion of CO_2_ through the porous layer of the already formed carbonate product.
(3) Chemical Reaction: The interfacial chemical reaction at the surface of the unreacted core.

The carbonation reaction has a fast rate in the initial stage followed by a very slow phase controlled by internal diffusion [56].

Direct dry carbonation requires no additional chemicals, but the reaction proceeds slowly under natural conditions [3]. It is essential to accelerate the reaction rate under high temperature, high pressure, combined with pretreatment methods like mechanical activation to reduce particle size and increase the specific surface area of SS [52,53,59,60]. These conditions can enhance reaction kinetics but lead to high energy consumption. In addition, a dense carbonate layer prevents the CO_2_ diffusion from reaching the unreacted core, with a potential risk of surface passivation [3]. Reactors such as fluidized beds are proposed to overcome heat and mass transfer limitations [61].

### 3.2. Direct Wet Carbonation

Direct wet carbonation refers to the reaction between SS and CO_2_ gas in a liquid environment. The water, as a solvent and transport medium, promotes the leaching of cations from the slag and the precipitation of carbonates. The carbonation reaction mainly involves three steps [62,63]:

(1) Dissolution and ionization of CO_2_: CO_2_ gas dissolves in water to form carbonic acid, which subsequently dissociates into bicarbonate ion and carbonate ions, lowering the pH of the solution. The reaction equation is shown in Equation (11).
(2) Dissolution of calcium and magnesium ions: the acidic environment generated by CO_2_ dissolution promotes the leaching of calcium and magnesium ions from the SS mineral phase into the aqueous solution. The reaction equation is shown in Equation (12).
(3) Carbonate precipitation: When the concentration of Ca^2+^/Mg^2+^ and CO_3^2−^_ ions in the solution exceeds the solubility product K_sp_ of their corresponding carbonates, nucleation and crystal growth occur, and ultimately form solid calcium carbonate and magnesium carbonate. The reaction equation is shown in Equation (13). When magnesium ions and calcium ions are present simultaneously, the product will form not only calcium carbonate and magnesium carbonate but also a portion of magnesian calcite.

CO_2_(g) + H_2_O(l) ↔ H_2_CO_3_(aq) ↔ H^+^(aq) + HCO_3^−^_(aq) ↔ 2H^+^(aq) + CO_3^2−^_(aq) (11)

Ca/Mg-silicates (s) + H^+^ (aq) → Ca^2+^/Mg^2+^ (aq) +SiO_2_ (s) + H_2_O (aq) (12)

Ca^2+^/Mg^2+^ (aq) + CO_3^2−^_ (aq) → (Ca/Mg)CO_3_ (s) (13)

The carbonation rate is affected by complex factors, including the rate of CO_2_ dissolution, the rate of Ca^2+^/Mg^2+^ dissolution, the rate of diffusion of CO_2_ gas and Ca^2+^/Mg^2+^ through the product shell layer, and the rate of carbonate precipitation. The carbonation reaction in the early stage is mainly affected by the dissolution rate of Ca^2+^/Mg^2+^ and the dissolution rate of CO_2_ gas. Previous studies have shown that the dissolution rate of Ca^2+^/Mg^2+^ for different minerals in SS varies greatly, which leads to a large difference in the carbonation conversion of SS with different chemical and mineral compositions [20,64]. Carbonate and other products generated are deposited on the surface of unreacted SS, which slows down the diffusion of carbonate ions and calcium ions, and decreases the reaction rate in the later stage of the reaction [65].

The main advantage of wet processes compared to dry processes is faster reaction rates at lower reaction temperatures (<100 °C) and ambient pressures [66,67]. However, challenges include high water consumption, the need for solid–liquid separation of the final products, and potential passivation of slag particles by the precipitating carbonates. Process enhancement techniques, such as high-shear reactors or additives that modify carbonate crystal growth, are a hot area of current research.

### 3.3. Indirect Carbonation

Indirect carbonation decouples the leaching and carbonation steps into a multi-stage process to overcome the limitations of direct routes. The typical indirect route involves [68]:

(1) Leaching: The slag is treated with a leaching agent (such as an acid or salt solution) to selectively extract calcium and/or magnesium into the solution, leaving behind a silica-rich residue.
(2) Carbonation: The Ca/Mg-rich leachate after separating from the solid residue is reacted with CO_2_ in a separate reactor to precipitate high-purity carbonates [52].

There are differences in pH requirements between the mineral leaching and carbonate precipitation stages. The leaching of the alkaline minerals needs to occur under acidic conditions, and a low pH needs to be maintained in the first stage. However, CO_2_ has a low solubility in acidic media, and an alkaline environment with a high pH needs to be provided in the precipitation stage [69]. Due to this characteristic, indirect mineralization processes usually use pH-swinging to optimize the mineralization efficiency [70].

Indirect carbonation achieves precise control of reaction conditions by separating the leaching and carbonation steps, and selectively extracts target components to reduce impurity effects and produce high-purity calcium carbonate. Studies have shown that calcium carbonate with a purity of up to 95% or more can be obtained by carbonation of SS leachate, which can be used in high-value-added fields such as papermaking, plastics and coatings [71]. However, this process is complex and requires additional chemical reagents, which increases economic costs. Efficient regeneration and recycling of solvents are necessary to achieve economic viability.

### 3.4. Microbial Carbonation

Microbial carbonation is an innovative technology that utilizes specific microorganisms or their metabolites to accelerate the carbonation reaction [72,73]. There are two main microbial mechanisms.

(1) Carbonic anhydrase: This enzyme is a metalloenzyme widely found in organisms that catalyzes the reaction of CO_2_ combining with water [74,75]. It can increase the hydration rate of carbon dioxide 10^7^ times [76]. Microorganisms reduce the time required for carbon fixation by carbonic anhydrase in microbial synergistic carbonation.
(2) Urea hydrolysis: Urease enzymes produced by specific bacteria hydrolyze urea to produce ammonia and carbonate ions. The production of ammonia raises the local pH and promotes the formation of carbonate precipitation from Ca^2+^ ions leached from the slag [77,78].

Microbial carbonation can be carried out at ambient temperature and pressure, which greatly reduces energy consumption and operating costs. However, the process is typically slower than abiotic chemical routes. Challenges include maintaining active microbial cultures, nutrient costs and potential contamination issues. Research in this area is still at an early stage, but it provides the potential for developing low-energy carbonation technologies.

## 4. Influencing Factors Steel Slag Carbonation Efficacy

The theoretical maximum CO_2_ sequestration capacity of SS can be calculated based on its chemical composition using stoichiometry. For example, 1 kg of pure CaO can theoretically sequester 0.785 kg of CO_2_. This implies 278–404 kg of CO_2_ can be theoretically sequestered for each ton of SS. However, the actual achievable carbonation conversion of SS is often lower and depends on the reaction pathway and process conditions.

### 4.1. Mineral Phase Composition

The carbonation activity of SS depends on its mineral composition [79]. Not all calcium-containing phases in SS have the same carbonation activity. Mineral compositions in SS are roughly divided into three categories: (1) f-CaO and Ca(OH)_2_; (2) calcium silicate minerals, such as C_2_S and C_3_S; (3) calcium ferrate or calcium ferroaluminate minerals, such as C_2_F and C_4_AF [13]. Different minerals have different degrees of carbonation reaction. Among these three types of minerals, f-CaO and Ca(OH)_2_ have high leaching reactivity and rapid carbonation in the initial stage of the reaction. They release Ca^2+^, which contributes to the alkalinity of the solution, accelerating the dissolution of carbon dioxide and the formation of carbonate ions. Calcium silicate minerals have slightly lower reactivity than f-CaO and Ca(OH)_2_ and also have high leaching reactivity. They are converted to soluble hydrated calcium silicate when reacting with water. A calcium-poor, silica-rich layer formed after releasing calcium ions, along with calcium carbonate deposited on the surface of particles, may act as a partial diffusion barrier, depending on its porosity and continuity, thereby impeding further ion transport in some systems. Calcium ferrate or calcium ferroaluminate minerals have little contribution to the carbonation reaction. Because they have difficulty in releasing Ca^2+^ to participate in the carbonation reaction and contribute less to the carbonation rate than the first two minerals [13]. The content of reactive phases like f-CaO, Ca(OH)_2_, C_2_S and C_3_S is the primary determinant of the carbonation capacity of SS. SS with higher proportions of these minerals exhibits higher carbonation activity and CO_2_ uptake. How to improve the carbonation rate of low reactive minerals has become one of the hot spots in current research.

### 4.2. Particle Size

Particle size is one of the most critical physical parameters affecting the carbonation rate of SS, which is usually limited by the dissolution rate of SS minerals [80,81]. The reduction in particle size can significantly improve the carbonation conversion of SS [82,83,84]. First, grinding slag into finer particles can increase the specific surface area of SS, providing more reactive sites and accelerating the leaching of Ca^2+^ ions from the mineral phase to the solution, thereby increasing the overall reaction rate. Second, a passivation layer of CaCO_3_ and silica gel forms on the surface of the slag particles. The distance for ions to cross the passivation layer to reach the core is shortened in fine particles, thus mitigating the inhibitory effect [53]. Third, the milling process disrupts the mineral crystal structure, exposing more active surfaces and forming structural defects that further enhance the reactivity. In addition, the strength of the Ca-containing phase is the weakest among the slag particles, and Ca tends to concentrate in the smaller slag particles. The CaO contents of SS with particle sizes of 840–1190 μm, 500–840 μm, 350–500 μm, 125–350 μm, and <125 μm were 43.59%, 45.3%, 46.49%, 47.24% and 48.16%, respectively, which reflected the tendency of calcium content to increase with decreasing particle sizes [62]. Studies have shown that the carbonation conversion increased from 24% to 74% when the particle size was reduced from <2 mm to <38 μm [65]. However, overfine milling not only increases energy consumption but also may lead to particle agglomeration, which in turn reduces the reaction efficiency [13]. Therefore, it is necessary to determine the optimal fineness of SS in practical applications. Controlling SS particle size within the range of approximately 38–150 µm typically enhances its specific surface area and reactivity, thereby achieving optimal carbonation conversion in reaction kinetics [2,42,67,85,86,87]. However, further reducing particle size substantially increases grinding energy consumption, and its economic viability requires comprehensive evaluation in combination with specific process conditions.

### 4.3. Temperature

In dry mineralization, higher temperatures (>300 °C) increase the reaction rate according to the Arrhenius equation [55,88]. However, temperature exhibits dual effects in aqueous systems, simultaneously influencing both reaction thermodynamics and kinetics. It alters the solubility of SS, CO_2_, and calcium carbonate while also affecting the thermodynamic constant and rate constant of the reaction. On one hand, elevated temperatures promote the dissolution of calcium and magnesium minerals in SS, which is typically diffusion-controlled. Increasing temperature enhances the effective diffusion coefficient of ions, thereby accelerating the release of reactants and increasing Ca^2+^ concentration in the aqueous solution [13,59,89]. On the other hand, an increase in temperature reduces CO_2_ solubility in water, decreasing the concentration of carbonate ions and weakening the driving force for carbonate precipitation [90].

These two opposing effects result in an optimal temperature for carbonation reactions. Below this temperature, the reaction is constrained by the slow dissolution kinetics of slag minerals; above it, the reaction is limited by reduced CO_2_ solubility. Most studies on SS aqueous carbonation indicate an optimal temperature range of 50 °C to 100 °C. The specific optimum value depends on other parameters, particularly CO_2_ pressure. In atmospheric or low-pressure systems like slurry reactors, this range typically falls around 50–60 °C [58,91]. In high-pressure reactors like autoclaves, where pressure compensates for CO_2_ solubility decline, the optimum temperature shifts upward to 100 °C [60,67]. Thus, the actual optimum temperature strongly depends on reactor type, system pressure, and the physicochemical properties of SS.

Furthermore, temperature significantly regulates the crystalline composition, microstructure, and growth location of calcium carbonate products [53]. At lower temperatures (10–20 °C), the liquid phase contains higher dissolved CO_2_ concentrations, leading to calcium carbonate formation primarily on slag particle surfaces or within the liquid phase [6,92]. Products predominantly consist of calcite and aragonite. At higher temperatures (60–80 °C), calcium carbonate dominated by aragonite is more easily formed at the gas–liquid interface and may create a dense product layer on slag surfaces, thereby influencing reaction depth. Practical operations require an appropriate temperature based on target products and process conditions while balancing energy consumption and economics. For instance, utilizing waste heat from steel factories can promote carbonation while reducing energy consumption.

### 4.4. CO_2_ Pressure

The partial pressure of carbon dioxide is a key parameter influencing the carbonation rate of SS [92]. In dry systems, high pressure increases the CO_2_ concentration at the particle surface; in wet systems, it enhances the solubility of CO_2_ in the aqueous phase according to Henry’s Law. Increasing CO_2_ partial pressure not only significantly enhances the amount of CO_2_ available in the solution to form carbonic acid, accelerating calcium carbonate precipitation, but also lowers slurry pH, promoting dissolution of alkaline minerals in SS [28,93]. When CO_2_ mass transfer becomes the rate-limiting step, pressurization effectively overcomes gas–liquid mass transfer constraints, accelerating reaction rates [13,53].

Increasing CO_2_ pressure from atmospheric pressure to 2–3 MPa substantially increases both the carbonation reaction rate and efficiency. For instance, increasing pressure from 0.1 MPa to 1 MPa doubles the carbonation conversion within the same reaction time [33]. However, CO_2_ pressure exhibits a distinct threshold effect on carbonation conversion similar to particle size. Below this threshold, increasing CO_2_ pressure substantially enhances carbonation rates. Beyond the threshold, its contribution becomes negligible. Excessive pressure may even accelerate carbonate formation, clogging particle pores and hindering further ion dissolution [94]. The specific value of this threshold is influenced by multiple factors, including temperature, SS characteristics, and reactor [65]. The threshold is approximately 10 bar at 50 °C but drops to 5 bar at 100 °C with 40% CO_2_, indicating that the CO_2_ pressure threshold decreases with increasing temperature [86].

Although high pressure can significantly enhance carbonation rates and calcium conversion, large-scale industrial processes require higher operational costs for gas compression in pressurized reactors and compressors. Notably, increasing the total pressure is necessary to ensure an effective CO_2_ partial pressure when using low-concentration flue gas. Under sufficiently long reaction times, the difference in final carbon fixation between different pressure conditions may decrease, and even low-pressure, long-term reactions may have higher carbon fixation than high-pressure, short-duration reactions. Therefore, methods such as alternating pressurization can be employed to reduce the cost of carbonation technology.

### 4.5. CO_2_ Concentration

The concentration of CO_2_ in the gas is closely related to CO_2_ pressure. Under constant total pressure, higher CO_2_ concentration results in greater CO_2_ partial pressure, which enhances the driving force for carbonation. Consequently, using a gas containing 40% CO_2_ leads to a significantly slower kinetics than using pure CO_2_ gas at the same total pressure [86]. However, slower kinetics may mitigate potential passivation, improving long-term conversion. Directly utilizing flue gas from steel mills eliminates the energy-intensive steps of CO_2_ capture and purification. However, the low CO_2_ concentration represents a slower reaction rate, which must be enhanced by increasing total pressure, raising the temperature, or further reducing SS particle size. A lower CO_2_ concentration can be employed during the initial mineralization stage to prevent excessive carbonation products from clogging gas transport pathways. Subsequently, increasing CO_2_ concentration in the later stages accelerates CO_2_ penetration into particles, thereby enhancing mineralization conversion. Nevertheless, impurities in flue gas such as SO_2_ and NO_x_ may interfere with the carbonation process, and their effects require further investigation.

### 4.6. pH

The pH of the slurry is a dynamic change as the mineralization reaction proceeds. When SS is mixed with water, the dissolution of CaO, Ca(OH)_2_, and calcium silicates releases a large amount of hydroxide ions, causing the initial slurry pH to rise to between 11 and 12.5. With the continuous introduction of CO_2_, the resulting carbonic acid continuously neutralizes OH^−^, leading to a gradual decrease in pH [95]. Acidic or neutral pH favors slag dissolution, while alkaline pH promotes carbonate precipitation. The dominant species of dissolved CO_2_ is carbonate ions at pH > 10. In the pH range of 6.4 to 10.3, the bicarbonate ion dominates. Dissolved CO_2_ is the main species when the pH is below 6.4. It is crucial to adjust the pH to achieve efficient carbonation [96]. Direct carbonation processes can be further accelerated by controlling the CO_2_ feed rate or implementing staged injection to optimize pH. In indirect or enhanced carbonation processes, alkaline substances like NaOH are often added to maintain pH within an optimal range, promoting carbonate ion formation and accelerating the carbonation reaction [97]. However, pH affects the leaching behavior of elements like Cr, V, and Mo, thereby influencing the environmental safety of the final product [33,66]. Thus, rational pH control is not only a key to enhancing carbonation conversion but also a critical measure for ensuring the long-term environmental stability of carbonation products.

### 4.7. Reaction Time

Reaction time is a critical parameter determining the carbonation rate of SS, exhibiting distinct phased characteristics [53]. The reaction rate is typically fast initially and then slows down significantly as the most reactive phases are consumed and passivation effects become more prominent. During the initial stage (10–30 min), highly reactive components such as f-CaO and partial calcium silicate rapidly react, driving a swift increase in carbonation conversion [91,98,99,100]. High reactant concentrations and low mass transfer resistance cause the system pH to rapidly decrease from its initial strongly alkaline state, creating favorable conditions for ion leaching. However, the reaction gradually shifts from being kinetics-controlled to diffusion-controlled as calcium carbonate and silica gel layers form, resulting in a rate slowdown [21,84].

Extending reaction time enhances carbonation conversion and promotes the transformation of calcium carbonate crystals into stable calcite phases. However, the effectiveness of this approach in improving carbonation conversion diminishes progressively. Excessive reaction times substantially increase reactor volume and investment. The optimal reaction time is an economic decision, balancing carbonation conversion and operational energy consumption. Under optimized conditions, maintaining reaction times between 30 min and 2 h typically achieves 70–80% of the carbonation conversion maximum [33]. Additionally, wet grinding can mitigate the effects of passivation layers and prolong the duration of high reaction rates.

### 4.8. Stirring Rate

Stirring is a critical operation in the wet carbonation process to overcome mass transfer limitations, enabling efficient mixing within the gas–liquid–solid three-phase system. First, agitation promotes gas–liquid mass transfer. The turbulence generated breaks large CO_2_ bubbles into smaller ones, increasing the gas–liquid interfacial area; simultaneously, it reduces the thickness of the liquid film retained at bubble surfaces, enhancing the dissolution rate of CO_2_ into water. Then, agitation ensures uniform suspension of slag particles in the slurry, preventing sedimentation. This maximizes the exposed solid reaction surface area and reduces the liquid boundary layer thickness around particle surfaces, which facilitates the migration of reactants like H^+^ and HCO_3^−^_ toward particle surfaces, as well as the transport of dissolved products Ca^2+^ into the bulk liquid phase. Furthermore, strong shear forces erode the passivated product layer forming on particle surfaces, exposing fresh unreacted interfaces to maintain a high reaction rate.

Typical laboratory studies use mechanical stirrers equipped with flat or inclined blades, or magnetic stirrers with magnetic stir bars, to agitate the slurry. The carbonation rate generally increases with the stirring rate up to a certain point. Studies indicate that below 500 rpm, increasing speed significantly enhances carbonation rate [65]. Beyond this threshold, reaction rates improve negligibly while mixing energy consumption rises continuously. Simultaneously, high stirring rates may alter the crystalline composition of calcium carbonate, such as favoring the formation of aragonite over calcite at 400–600 rpm. Typical laboratory studies use stirring speeds in the range of 300 to 800 rpm. For industrial scale-up, optimizing stirring requires mixing parameters like power input per unit volume and impeller tip speed to achieve the optimal balance between enhanced mass transfer and energy consumption.

### 4.9. Liquid-to-Solid Ratio

Liquid-to-solid ratio (L/S) represents the proportion of water to slag in the wet carbonation, directly influencing the rheological properties of the slurry, reactant concentration and mass transfer. A low L/S enhances slurry alkalinity and ionic concentration, promoting calcium carbonate precipitation primarily on SS particle surfaces. It also reduces the amount of water that needs to be heated, pumped, and later removed from the products and subsequent treatment energy consumption. However, its drawback lies in excessive slurry viscosity that is difficult to sufficiently mix, potentially leading to poor gas dispersion, inadequate particle suspension, and restricted mass transfer, thereby diminishing carbonation efficiency. Conversely, a high L/S creates a low viscosity slurry conducive to mixing and mass transfer. Yet it significantly dilutes calcium ion concentration in the liquid phase, weakening the driving force for calcium carbonate precipitation. Capillary adsorption of water in particle pores impedes CO_2_ diffusion. Furthermore, this also improves equipment and water usage costs. Therefore, selecting L/S requires balancing mass transfer and reaction driving forces. The optimal L/S range is 3–20 L/kg [58,65,85,99], with specific values determined by factors such as SS particle size, mineral composition, and reactor type. Finer particles form higher viscosity slurries under the same L/S, requiring an appropriately increased L/S.

### 4.10. Reaction Equipment

SS carbonation reaction equipment is primarily categorized into autoclave, water bath reactor, fluidized bed, and rotating gravity bed [100,101]. Autoclaves provide high-temperature, high-pressure environments that effectively promote CO_2_ dissolution and ion mass transfer, but with high energy consumption [13,100]. Fluidized bed reactors enable continuous processing, high rates of heat and mass transfer, and uniform temperature distribution [101]. However, their design and operation are more complex, and particle attrition can be an issue. Water bath reactors involve mixing SS powder with liquid to form a slurry before introducing CO_2_ [91]. These reactors are simple to operate and have lower energy consumption. High-gravity rotating packed beds utilize centrifugal force to create a high-gravity environment to enhance mass transfer and partially disrupt the product layer, significantly increasing reaction rates [98,99]. The selection of reactors influences the process parameters for SS carbonation, which is central to achieving efficient carbonation.

### 4.11. Leaching Solvent Type

While water is the universal and most economical solvent, the carbonation rate can be enhanced by using alternative solvents or additives, and its effect mainly depends on the solvent type and concentration. There are many types of solvents, such as strong acids (HCl, HNO_3_, H_2_SO_4_), weak acids (CH_3_COOH), ammonium salts (NH_4_Cl, NH_4_NO_3_, (NH_4_)_2_SO_4_, NH_4_HSO_4_, CH_3_COONH_4_) and so on [102,103,104,105,106,107]. Strong acids can efficiently extract calcium from SS with leaching rates of more than 90%, but their strong corrosiveness, high cost and energy consumption for subsequent regeneration limit their industrial application [53,108]. In contrast, weak acids and ammonium salts have attracted more attention because of their low corrosiveness and recycling potential. Acetic acid selectively leaches calcium and regenerates it after carbonation through a “pH swing” process. Ammonium solutions provide an acidic environment during the leaching phase and use the released ammonia to form an alkaline buffer system during the carbonation phase. It spontaneously completes the pH adjustment without the need for additional acid or alkali additives, thus allowing for solvent recycling. In addition to acidic additives, sodium salts (NaHCO_3_, NaCl) and magnesium salts (MgCl) can also promote dissolution of the calcium-silica phase by changing ionic strength or homoionic effects [109,110,111]. Strong bases (NaOH) enhance carbonate ion concentration by creating a high alkalinity environment [68].

### 4.12. Relationship Among Different Parameters

The aforementioned influencing factors are not independent variables. They exhibit complex interrelationships, where changing one parameter may trigger chain reactions affecting the optimal settings of others. For instance, the optimum temperature increases with rising CO_2_ pressure because higher pressure counteracts the negative effect of temperature on CO_2_ solubility. Finer particles require more vigorous agitation to maintain suspension and overcome high slurry viscosity. Lower L/S increases slurry viscosity, necessitating higher stirring rates. This complexity demands a multivariate approach rather than sequential single-factor adjustments to optimize the process. Statistical methods such as response surface methodology or factorial design of experiments can be employed to establish empirical models predicting the functional relationship between carbonation conversion and variables. This enables maximizing CO_2_ uptake while minimizing costs.

## 5. Industrialization Demonstration of SS Carbonation

SS carbonation technology is an important path to achieve industrial solid waste resource utilization and carbon emission reduction. There are a number of industrialization demonstration projects around the world.

In China, Jingyun Taibo New Material Science and Technology Co., Ltd. in Shandong put into a 10,000-ton CO_2_ direct carbonation project in August 2023 [112]. SS, fly ash, carbide slag and other solid waste react with CO_2_ flue gas to prepare high-strength carbon-negative building materials. Each ton of building materials can fix 0.3–0.5 tonnes of CO_2_. The project treats 160,000 tonnes of solid waste, produces 400 square meters of carbon-sequestering stone building materials, and reduces CO_2_ emissions by more than 60,000 tonnes annually. The University of Science and Technology Beijing completed a 1000-ton industrial demonstration of SS carbonation in the HISCO Group in 2024, achieving the use of SS and direct in situ fixation of industrial flue gas [113]. Zhejiang University and Henan Qiangnai New Material Co., Ltd. realized an industrial pilot experiment of CO_2_ deep carbonation curing for building materials in Henan in 2020, using pure CO_2_ curing instead of traditional steam curing [114]. During the 72-h trial operation of the system, a total of 101 tonnes of CO_2_ were injected, and the residual CO_2_ was 1.04 tonnes, with a CO_2_ conversion rate of 98.97%. Shanxi University has established a 50,000-ton SS mineralization industrial demonstration, forming f-CaO treatment technology in SS micropowder that meets the national standard requirements [115]. Baosteel Group adopted the SS carbonation method of Columbia University and completed the first-stage project in 2021 [114]. The project can deal with 100,000 tonnes of SS and 20,000 tonnes of carbon dioxide per year and produce 40,000 tonnes of high-purity calcium carbonate and 70,000 tonnes of iron-containing products per year. Shanxi Institute of Coal Chemistry, Chinese Academy of Science, has completed a hundred tonnes of SS indirect carbonation pilot test by using self-developed high-efficiency leaching agent, obtaining CaCO_3_ with 98.88% purity and 90.53% whiteness [116]. In addition, the original initial science and technology demonstration project adopted wet indirect mineralization of carbide slag using NH_4_Cl as the leaching agent [117]. The calcium chloride reacts with ammonia and CO_2_ to generate CaCO_3_, and the generated NH_4_Cl solution was recycled. During the whole process, the flue gas of the power plant does not need to be captured and purified, and the recycling medium solution can be used repeatedly. A thousand-ton demonstration in the Datong Power Plant of Guodian Shanxi has been built and put into production.

The JFE company in Japan has developed SS thermal crushing technology. Carbon dioxide was injected into high-temperature SS to achieve rapid carbonation, while the carbonation gas heat was recovered to improve energy efficiency, and carbonated SS was used for road aggregates [118]. CarbiCrete in Canada implemented an industrial demonstration of carbonated SS concrete with an annual carbon sequestration capacity of 8000 tonnes in 2020, by replacing cement with SS, and mixing and hardening with CO_2_ instead of curing steam [119,120]. Aalto University in Finland constructed the world’s first test facility of mineral carbonation in 2014, using NH_4_Cl as a leaching agent to produce precipitated calcium carbonate [121]. The pilot plant in batch mode can process up to 20 kg of SS and 190 L of liquid solvent and produce approximately 10 kg of calcium carbonate. The Ca^2+^ leaching rate in the slag was 80%, the CO_2_ conversion rate was 71%, and the purity of the prepared CaCO_3_ was 99.5%.

Generally, SS carbonation technology is gradually moving from the laboratory to large-scale applications. Although there are still challenges in the reaction rate, energy consumption and cost control, the technology has shown significant carbon emission and resource utilization potential through several industrial demonstration projects in China and abroad.

## 6. Environmental Benefits and Economic Evaluation

Life cycle assessment (LCA) is one of the internationally recognized methods for systematically evaluating the environmental performance of technologies [122,123]. It is necessary to comprehensively consider the resource consumption and environmental impacts of each step in the carbonation process of SS collection and treatment, CO_2_ capture and transportation, carbonation reaction, product use and disposal. The comparison between SS carbonation technology and traditional SS treatment methods can fully reveal the environmental advantages and potential problems of SS carbonation [124].

Although SS carbonation can achieve CO_2_ emission reduction and solid waste resource utilization, its overall environmental benefits are significantly constrained by factors such as pretreatment energy consumption and reaction conditions. Methods such as increasing the specific surface area of SS, increasing the temperature and pressure, and increasing the CO_2_ concentration are usually adopted to improve the carbonation efficiency, but these measures will increase the energy consumption and carbon emission. LCA has shown that the environmental impacts of different carbonation routes vary. The dry pathway shows a significant increase in global warming potential (GWP) after extended carbonation time with limited increase in CO_2_ uptake. In contrast, the wet carbonation can approach the maximum CO_2_ uptake in a shorter period of time due to the large amount of water involved in the reaction, and the overall GWP impacts are typically lower than the dry method [124]. Sara et al. [125] compared the wet method with L/S of 0.3 dm^3^/kg and the slurry method with L/S of 5 dm^3^/kg for direct carbonation, and found that both GWPs were negative, but the wet method had approximately twice the impact of the slurry method. The main sources of GWP contribution differed in the two pathways, the wet method coming mainly from the carbonation, compression, grinding and transportation, and the slurry method being more affected by CO_2_ compression, mixing, grinding and transportation. The direct use of CO_2_-rich flue gas can replace the compression step and significantly reduce energy consumption and emissions. In addition, the indirect carbonation also needs to optimize the reaction parameters and utilize flue gas [126]. Li et al. [127] have shown that the two biggest contributors to the GWP are electricity consumption for carbonation curing and the production of CO_2_ gas cylinders, and the carbonation curing accounts for 74% and 20% of the total emissions for SS aggregate and SS blocks, respectively.

The number of LCA studies on SS carbonation is relatively few, and the methods of evaluation studies are different, which makes it difficult to compare directly. In the future, a unified, universal LCA assessment framework and standards of SS carbonation processes need to be established to cover the entire process from raw material processing, carbonation reactions and product utilization, focusing on the GWP evaluation. Furthermore, it is suggested that machine learning be applied for data integration and process simulation to optimize the environmental impact of process pathways.

Studies have shown that indirect carbonation produces high-purity calcium carbonate, and it can improve the economics [128]. Lee et al. [129] showed that the production cost of nano-calcium carbonate is about 484 USD/ton, which is competitive to the current production cost of commercial calcium carbonate (400–500 USD/ton). The highest capital cost is electrolysis equipment, and the highest operating cost is the high electricity consumption for NaCl electrolysis. In addition, the use of carbonated SS as a cementitious material can also be economically valuable, with an economic yield of 16.5 CNY/ton SS and GWP of 24.9 kg CO_2_-eq/ton SS [130].

In conclusion, the life cycle assessment and economic benefit analysis together show that SS carbonation technology has both environmental and resource synergistic benefits. The future development should focus on optimizing the energy structure, reducing the power consumption of the key links, and developing high-value-added products.

## 7. Conclusions and Prospects

This paper reviews the current research status of SS carbonation, including the mechanism, influencing factors, industrial application and environmental impact. The following main conclusions can be drawn.

SS carbonation exists in a variety of methods. Direct dry carbonation is suitable for conditions of sufficient heat, direct wet carbonation is the most mature, the indirect method has the potential to produce high-value-added products, and microbial carbonation technology is under development.

SS carbonation reaction is a multi-phase complex process, which is affected by various factors such as mineral phase, particle size, reaction temperature, CO_2_ pressure, CO_2_ concentration, pH, reaction time, stirring rate, liquid-to-solid ratio, reaction equipment, solvent type and so on. The presence of water can significantly reduce the reaction activation energy and improve the carbonation conversion. The mineral composition determines the theoretical capacity, in which f-CaO and C_2_S exhibit the highest mineralization activity. Particle size reduction generally increases the reaction rate, but grinding energy consumption needs to be considered. Temperature varies by reaction pathway. Carbon dioxide pressure and concentration affect CO_2_ solubility in the aqueous phase. A pH of 8–11 favors carbonate precipitation but limits mineral dissolution. Stirring promotes mass transfer, but threshold effects are seen above 500 rpm. Liquid-to-solid ratio and reaction time need to be optimized to avoid dilution and passivation, respectively. Reactor design needs to match the kinetics of the reaction path. The choice of leaching solvent in indirect methods needs to take into account the regeneration cost, and NH_4_Cl is a good choice between efficiency and sustainability.

At present, several industrialization demonstration projects of SS carbonation technology have been built worldwide. Carbonation technology has obvious advantages from the perspective of environmental and economic benefits. In conclusion, SS carbonation technology, as one of the important paths for the steel industry to realize green transformation and the “double carbon” goal, has a broad development space in the future.

Despite significant achievements in SS carbonation technology, numerous challenges remain in its large-scale industrial application, requiring continuous in-depth research.

(1) At the technical level, the current challenge of SS carbonation technology lies in balancing reaction rate and energy consumption. Due to the complexity of the mineralization process and its numerous variables, machine learning can be employed to analyze large datasets related to mineralization. This can identify intricate correlations between process parameters and outcomes, optimizing the process and enhancing mineralization efficiency. Developing machine learning models trained on multi-scale data from mineralogy to reactor hydrodynamics can enable predictive optimization to maximize efficiency under fluctuating feedstock conditions. New reactors such as fluidized beds and supercritical carbon dioxide reactors can also be developed to maximize mass transfer efficiency, thereby reducing reactor size and lowering energy consumption. Reactors should focus not only on mass transfer but on their integration with steel plant infrastructure, including direct use of low-grade waste heat and low-concentration flue gas. Meanwhile, low-energy slag-selective activation methods, including mechanical grinding, thermal treatment, or chemical activation, should be explored to enhance less reactive mineral phase reactivity instead of broad energy-intensive activation. Furthermore, microbial synergistic interactions and enzymatic catalysis mechanisms should be researched to optimize carbonation processes. Rather than studying microbial carbonation in isolation, a promising yet underexplored avenue is coupling microbial pretreatment to alter slag surface chemistry with subsequent accelerated chemical carbonation.
(2) In terms of economy, the investment cost of SS carbonation technology is high, and the product market competitiveness is insufficient. This technology requires additional investment in equipment and operation compared to traditional SS treatment methods. Costs can be reduced through process optimization, system integration and scale effects. The carbonation units can be integrated with the steel production process, utilizing SS and flue gas in the plant to avoid the logistical burden of large-scale material transportation. Simultaneously, the abundant low-grade waste heat resources from cooling processes in steel plants can provide energy for carbonation. This builds a closed-loop system in the plant, significantly reducing energy consumption and operational costs. In addition, high-added-value products are produced by controlling the properties of carbonate products. Utilizing carbonated SS as concrete aggregate or supplementary cementitious materials can be applied in civil engineering and road construction. Achieving the transformation from waste to high-value products would improve the economic viability of SS carbonation technology.
(3) The development of SS carbonation technology is inseparable from supportive policies and a robust standardization system. Currently, the technology faces problems such as unclear product classification, lack of standards and an imperfect certification system, which constrain its market acceptance. Policy guidance should be strengthened in the future. SS carbonation should be incorporated into carbon emission reduction accounting systems, and corresponding incentive measures should be established. At the same time, technical standards and evaluation systems for carbonated products are essential to standardize product quality and promote their entry into the market and widespread application.

The future of SS carbonation technology depends on the continuous innovation of the process, the steady development of the carbonated materials market and the implementation of climate policies. SS carbonation can truly achieve large-scale industrialization and make contributions to the green transformation of the steel industry and global carbon emission reduction targets through the synergistic promotion of technology, economy and policy.

## Figures and Tables

**Table 1:** Mineral phases of SS.

Element	Possible Mineral Phases
Ca, Si, Al	f-CaO [32,44,45], Ca(OH)_2_ [29,33,41,46], CaCO_3_ [33,46], β-C_2_S [33,35,47], γ-C_2_S [32,33], α-C_2_S [29,35], C_3_S [27,29,32,45,46], C_12_A_7_ [35,39,41]
Si, Ca, Al	SiO_2_ [45], CaAl_2_SiO_6_ [47], Ca_2_Al_2_Si_2_O_7_ [47], Ca_3_Al_2_(SiO_4_)_1.5_(OH)_0.5_ [33], Ca_4_Si_2_O_7_(F,OH)_2_ [35]
Fe, Ca, Al	FeO [32,33,46,47], Fe_2_O_3_ [46,47], Fe_0.9536_O [27], Ca_2_Fe_2_O_5_ [27,33,46], Ca_2_(Al,Fe)_2_O_5_ [47], Ca_2_Fe_1.01_4Al_0.986_O_5_ [29,45], C_4_AF [32,44]
Mg, Fe, Si, Ca, Al	f-MgO [32], Mg(OH)_2_ [39], MgFe^3+^_2_O_4_ [33], Mg_0.239_Fe_0.761_O [46], (MgO)_0.593_(FeO)_0.407_ [32], Mg_2_SiO_4_ [35], (Mg,Fe)_2_Si_2_O_6_ [35], Ca_7_Mg(SiO_4_)_4_ [33], CaMgO_6_Si_2_ [32], Ca_2_MgO_7_Si_2_ [32], CaMgSiO_4_ [35], Ca_3_Mg(SiO_4_)_2_ [33,35,47], MgAl_2_O_4_ [35]

**Table 2:** Characteristics, advantages and disadvantages of the carbonation route for SS.

Route	Characteristics	Advantages	Disadvantages
Direct dry carbonation	A single-step process where the SS and CO_2_ react directly in a dry environment.	Simple process and easy operation.	Under high-temperature and high-pressure conditions, the energy consumption of the reaction is high and the reaction equipment is stringent. Reaction rates are slow, and mineralization conversion is low.
Direct wet carbonation	A single-step process where the SS and CO_2_ react directly in a wet environment.	Simple reaction with relatively high reaction rates and mineralization conversion.	A large amount of water is required. Energy consumption is relatively high. Separating the product from SS is difficult.
Indirect carbonation	A two-step or multi-step process that first extracts calcium and magnesium ions from SS into solution, followed by a carbonation reaction with CO_2_.	High leaching efficiency and high-purity calcium carbonate production.	The process is complex and relatively costly.
Microbial carbonation	A technology that incorporates microorganisms into the mineralization process of SS.	Low energy consumption and high mineralization conversion.	Cultivating and maintaining microbial activity is difficult.

## Data Availability

No new data were created or analyzed in this study. Data sharing is not applicable to this article.
